# Regarding RECIST in retrospective studies

**DOI:** 10.1093/oncolo/oyaf182

**Published:** 2025-07-09

**Authors:** Lawrence W Wu, LawrenceH Schwartz, Susan E Bates

**Affiliations:** Division of Hematology/Oncology, Department of Medicine, Columbia University Irving Medical Center, New York, NY 10032United States; Department of Radiology, Memorial Sloan Kettering Cancer Center, New York, NY 10065, United States; Division of Hematology/Oncology, Department of Medicine, Columbia University Irving Medical Center, New York, NY 10032United States; Hematology/Oncology Research, James J Peters Veterans Affairs Medical Center, Bronx, NY 10468, United States

Retrospective studies in oncology can provide enormous value by capturing large amounts of clinically relevant data, highlighting populations and conditions that may not be well captured in clinical trials, and avoiding the financial costs associated with prospective clinical trials.^1,2^ However, the quality of these studies can vary, particularly if investigators attempt to approximate data from prospective studies.

Response Evaluation Criteria in Solid Tumors version 1.1 (RECIST v1.1) is widely accepted and utilized in clinical trials of solid tumors.^3,4^ In brief, with a maximum of 2 lesions per organ, the sum of the longest diameters (SLD) of up to 5 target lesions ≥10 mm in diameter is calculated.^5^ Nontarget lesions <10 mm and ascites or pleural fluid are considered evaluable and nonmeasurable. Intervals for imaging assessments are prespecified in clinical trial protocols. RECIST v1.1 response criteria are described in Table 1.^5^ Based on these response criteria, specifically with respect to progressive disease, surrogate endpoints such as progression-free survival (PFS) are identified. While not perfect, these criteria are standardized, objective, and well validated, ensuring reliability when applied rigorously in clinical trials.^4,6^

**Table 1:** RECIST v1.1 response criteria

Complete response	Complete disappearance of all lesions and pathologic lymph nodes
Partial response	≥30% decrease in SLD with no new lesions or progression of nontarget lesions
Progressive disease	≥20% increase in SLD OR new lesions OR progression of non-target lesions
Stable disease	Does not meet criteria for any of the above

Abbreviations: SLD, sum of the longest diameter of each target lesion.

However, the same degree of standardization cannot be reliably applied in real-world clinical practice and in most retrospective studies. Factors such as variable intervals of assessments, inconsistent anatomic coverage, and the use of diverse imaging modalities present challenges to the use of RECIST v1.1 in the real world.^7^ Biases due to toxicity and availability of the next therapy can make determining progression especially fraught. Additionally, clinical reads in the real world are often performed by different radiologists, often measuring different tumors with the assessment of response typically not scored in accordance with RECIST definitions. The application of RECIST in this manner is analogous to an attempt to retrospectively apply the Common Terminology Criteria for Adverse Effects based on clinical notes. It is impossible to think that a precise determination of toxicity grading can be obtained from clinical notes in a retrospective analysis.

Several authors have described “real-world” RECIST (rwRECIST) with different strategies taken to adapt RECIST. One study that attempted to retrospectively apply RECIST v1.1 criteria to real-world radiology reports found that RECIST was inadequate in assessing progression.^8^ While the addition of clinician-assessed response considerably improved progression assessment, discordance still occurred in up to 29% of cases.^7,8^ Similar studies concluded that clinician notes can provide a reasonable estimate of response.^9,10^ However, these cannot be conflated with RECIST responses.

Alternative endpoint assessments have been developed, including time to treatment discontinuation (TTD), and time to next treatment (TTNT), measured from time of initial treatment to time of treatment discontinuation for any reason, and time the next treatment started, respectively. TTD has been evaluated in large real-world datasets in nonsmall-cell lung cancer (NSCLC) and has demonstrated high concordance with PFS in clinical trials for NSCLC.^11^ As with “real-world PFS,” biases with this approach also include inaccurate assessment for fixed duration treatments and continuation of ineffective treatment if there are few subsequent alternatives. TTNT has also demonstrated high concordance with PFS in clinical trials for a variety of solid malignancies but may also be subject to similar biases.^12,13^ These endpoints necessarily include patients discontinuing for toxicity as well.

Seeing an increase in real-world analyses, in 2018, the United States Food and Drug Administration (USFDA) issued guidance of a framework for real-world evidence that could influence FDA regulatory decisions.^14^ This has been followed up with guidance documents from other regulatory agencies (Table 2). Follow-up guidance from the FDA has provided greater insight into how data from real-world registries, electronic health records, and medical claims should be reported to conform to higher standards that would support regulatory decisions on safety or effectiveness.^15,16^ Additional frameworks for real-world evidence have been provided by other regulatory agencies including the European Medicines Agency and the United Kingdom’s National Institute for Health and Care Excellence (NICE).^17,18^ These guidance documents emphasize defining methods of clinical data collection, identifying confounders, and ensuring the reliability of data. However, no particular methodology or response endpoints are favored by these guidance documents, and this remains an area of active investigation.

**Table 2.** Real-world evidence guidance

Issuing agency	Title	Year
FDA	Framework for FDA’s Real-World Evidence Program^14^	2018
FDA	Real-World Data: Assessing Registries to Support Regulatory Decision-Making for Drug and Biological Products^15^	2023
FDA	Real-World Data: Assessing Electronic Health Records and Medical Claims Data To Support Regulatory Decision Making for Drug and Biological Products (Draft Guidance)^16^	2021
EMA	Real-world evidence framework to support EU regulatory decision-making^17^	2023
NICE	NICE real-world evidence framework^18^	2022

Abbreviations: EMA, European Medicines Agency; FDA, Food and Drug Administration; NICE, National Institute for Health and Care Excellence;

There are several widely utilized guidelines for reporting observational studies and case reports (Table 3). The most widely used guideline for observational studies is the **St**rengthening the **R**eporting of **Ob**servational Studies in **E**pidemiology (STROBE).^19^ The **Re**porting of Studies **C**onducted using **O**bservational **R**outinely Collected Health **D**ata (RECORD) statement is an extension of the STROBE guidelines, specifically for data collected through electronic health records.^20^ The CAse REport (CARE) guidelines provide a standardized checklist for key elements of a case report.^21^ However, none of these guidance documents indicate a preference for a particular endpoint.

**Table 3.** Guidance for reporting of retrospective studies.

Reporting guideline	Type of study	Year
STROBE^19^	Cohort studies, case–control studies, cross-sectional studies, combined (observational studies)	2007
RECORD^20^	Cohort studies, case–control studies, cross-sectional studies, combined (observational studies using routinely collected health data)	2015
CARE^21^	Case report	2013

Abbreviations: CARE, CAse REport; RECORD, Reporting of Studies Conducted using Observational Routinely Collected Health Data; STROBE, Strengthening the Reporting of Observational Studies in Epidemiology.

Given this lack of specific guidance for endpoints, we would argue appropriately applied TTD and TTNT may more accurately reflect real-world outcomes in retrospective studies and should be strongly considered in lieu of RECIST v1.1-defined PFS. Ultimately, generating reliable retrospective data is paramount to forming rational hypotheses for future prospective clinical trials, as well as for making determinations about therapies already in the clinic.

## Data Availability

No new data were generated or analyzed in support of this research.
